# *Rickettsia parkeri* in *Amblyomma triste* from Uruguay

**DOI:** 10.3201/eid1008.030999

**Published:** 2004-08

**Authors:** José M. Venzal, Aránzazu Portillo, Agustín Estrada-Peña, Oscar Castro, Perla A. Cabrera, José A. Oteo

**Affiliations:** * Universidad de la República, Montevideo, Uruguay;; † Hospital de La Rioja, Logroño, Spain;; ‡ Universidad de Zaragoza, Zaragoza, Spain

**Keywords:** Amblyomma triste, Spotted Fever Group rickettsia, Rickettsia parkeri, Uruguay, ticks, rickettsioses, dispatch

## Abstract

Our goal was to detect whether spotted fever group *Rickettsia* are found in the suspected vector of rickettsioses, *Amblyomma triste*, in Uruguay. *Rickettsia parkeri* was detected in *A. triste*, which suggests that this species could be considered a pathogenic agent responsible for human rickettsioses in Uruguay.

In South America, cases of rickettsioses produced by the genus *Rickettsia* have been described in several countries in the last 20 years. The first three native cases of rickettsioses in Uruguay were reported in 1990. Patients had an initial small necrotic lesion (eschar) on the tick-bite point of attachment, fever and regional lymphadenopathies, an erythematous maculopapular rash, or any combination of these symptoms. Ticks involved in these cases were classified as *Amblyomma triste* (1), formerly thought to be *A. maculatum* (2).

*A. triste* is a neotropical tick species with a variety of hosts (3,4). It is the main tick species feeding on humans in Uruguay, and it is the primary candidate vector for transmitting rickettsioses in this country (5). According to the literature (2), *Rickettsia conorii* has been the causative agent of rickettsial diseases in Uruguay, but the evidence has been only serologic (by antirickettsial microimmunofluorescence testing) in all patients with suspected rickettsioses (6,7). Neither rickettsial isolation nor polymerase chain reaction (PCR) amplification from human blood samples from patients from Uruguay have been performed. However, as has been suggested (8), other tick-transmitted rickettsiae could be present in Uruguay.

## The Study

The aim of this study was to identify the spotted fever group (SFG) rickettsial species present in the suspected vector of SFG rickettsioses in Uruguay (*A. triste*). From 1999 to 2004, in Uruguay, ticks were collected from humans (with and without rickettsial syndrome), other mammals, and vegetation and preserved in ethanol 70% at room temperature. Species, sex, and stage of development were determined by members of the Facultad de Veterinaria, Universidad de la República (Uruguay). Classified adult ticks (N = 91) were sent to the Hospital de La Rioja (Spain) for analysis with molecular biologic techniques. Thirty-six ticks recovered from 14 humans were attached but nonengorged. Only one tick removed from a human, the one corresponding to human 3, was attached and engorged. A total of 16 *A. triste* were captured walking on three different humans (nonattached). The remaining ticks were attached to two goats (n = 3), a rodent of the species *Scapteromys tumidus* (n = 4), and three dogs (n = 30; 19 of them were engorged). One tick was recovered from vegetation. Details are shown in the Table.

DNA from the ticks was extracted by using the Tissue DNA Spin Kit (Genomed, Granada, Spain) according to the manufacturer's instructions. PCR testing for *ompA*, *gltA*, and 16S rRNA genes was performed as previously described (9–11). Two negative controls (one of them with template DNA but without primers and the other with primers and containing water instead of template DNA) as well as a positive control (*R. conorii* Malish #7 grown in Vero cells) were included in all PCR assays. Restriction analysis of *ompA* amplicons was also carried out under conditions reported by Roux et al. (12). Each PCR-amplified fragment of *ompA* gene was sequenced twice for all positive samples (Universidad de Alcalá de Henares, Spain) to confirm the identification of rickettsiae. Data were aligned with homologous sequences of reference strains of the SFG rickettsiae retrieved from the GenBank database.

Six ticks (three females and three males) collected on three humans and three dogs yielded positive PCR products of the expected sizes for *ompA*, *gltA*, and 16S rRNA, respectively (Table). One of these ticks infected with SFG *Rickettsia* (the only one that was engorged) was removed from a woman (human 3) diagnosed with rickettsial syndrome in the Instituto de Higiene, Facultad de Medicina, Universidad de la República (Uruguay). This patient showed a small initial maculopapulous lesion on her scalp at the tick-bite point, followed by regional lymphadenopathies and fever. Diagnosis was made on the basis of the clinical picture and indirect immunoglobulin (Ig) G immunofluorescent technique with *R. conorii* antigen (Biomerieux Laboratories, Marcy l'Etiole, France). Serum specimens were collected during the acute phase (day 0) and convalescent phase (1 month later). The patient showed seroconversion for *R. conorii* with IgG, and she had a benign disease course after treatment with oral tetracyclines. No clinical signs of infection were confirmed for the remaining two humans bitten by ticks infected with SFG *Rickettsia* (humans 6 and 7), but ticks were removed immediately after attachment in these cases. For all six positive samples, sequence analysis for *ompA* amplicons showed 100% similarity with the homologous sequence of *R. parkeri* (GenBank accession no. U43802). Profiles obtained with *Rsa*I for *ompA* PCR fragments were also in accordance with these data.

**Table:** *Amblyomma triste* ticks collected from different origins in Uruguay^a^

Host	No. of ticks (N = 91)		Location	Date of isolation	PCR amplification			
	Males	Females			*ompA*	*gltA*	16S rRNA	SFG *Rickettsia* species found in ticks
Human 1	0	1	Maldonado	Nov 1999	-	-	-	
Human 2	2	0	Canelones	Oct 2000	-	-	-	
Human 3^b^	1	0	Montevideo	Oct 2000	+	+	+	*R. parkeri*
Human 4	2	0	Maldonado	Dec 2000	-	-	-	
Human 5	1	0	San José	Oct 2001	-	-	-	
Human 6	0	1	Canelones	Sep 2002	+	+	+	*R. parkeri*
Human 7	1	0	Montevideo	Dec 2002	+	+	+	*R. parkeri*
Human 8	1	2	Montevideo	Oct 2002	-	-	-	
Human 9	2	7	Montevideo	Oct 2002	-	-	-	
Human 10	1	1	Canelones	Aug 2003	-	-	-	
Human 11	1	1	Canelones	Aug 2003	-	-	-	
Human 12	2	1	Montevideo	Oct 2003	-	-	-	
Human 13	0	1	Montevideo	Sep 2003	-	-	-	
Human 14	3	4	Canelones	Sep 2003	-	-	-	
Human 15	4	8	Montevideo	Oct 2003	-	-	-	
Human 16	2	0	Canelones	Nov 2003	-	-	-	
Human 17	1	0	Canelones	Nov 2003	-	-	-	
Human 18	0	2	Montevideo	Jan 2004	-	-	-	
Goat 1	1	0	Maldonado	Nov 1999	-	-	-	
Goat 2	0	2	Canelones	Oct 2000	–	-	-	
Rodent	2	2	Montevideo	Oct 2000	-	-	-	
Dog 1	3	21	Maldonado	Dec 2000	+ (3)	+ (3)	+ (3)	*R. parkeri*
Dog 2	0	1	San José	Oct 2001	-	-	-	
Dog 3	1	4	Canelones	Sep 2002	-	-	-	
Vegetation	0	1	Montevideo	Dec 2002	-	-	-	

^a^PCR, polymerase chain reaction; SFG, spotted fever group. ^b^Human 3 had rickettsioses.

## Conclusions

SFG *Rickettsia* isolated from arthropods and initially classified as nonpathogenic to humans are increasingly recognized as causing emerging rickettsial diseases (13). In the last 10 years, different *Rickettsia* species and subspecies, such as *R. aeschlimannii* (14), *R. sibirica* strain *mongolotimonae* (15), and *R. slovaca* (16), among others, have been implicated as human pathogens. Very recently, a new tickborne *Rickettsia*, *R. parkeri*, has been identified as a cause of human disease in the southern United States (17). According to Paddock et al., *R. parkeri* rickettsioses may also occur in other regions of the Western Hemisphere, e.g., in Uruguay.

We report *R. parkeri* infection in *A. triste* ticks collected in Uruguay. Several cases of rickettsioses have been described in this country but, to date, no *Rickettsia* has been isolated, cultivated, and characterized as the causative agent. A few years ago, *R. conorii* was presumptively considered the etiologic agent, but diagnosis was established with serologic assays (indirect microimmunofluorescence testing) as reference technique (6). Cross-reactions are noted within SFG *Rickettsia* antigens, and available serologic tests cannot be used to implicate a specific pathogen. In Uruguay, *A. triste* frequently bites humans, and rickettsioses frequently develop in them (5). Our finding of *R. parkeri* infection in one *A. triste* tick collected from a patient with rickettsiosis suggests that *R. parkeri* could be a pathogenic SFG *Rickettsia* involved in rickettsial diseases in Uruguay. Traditionally, this agent was reported as nonpathogenic to humans, but the first report of a human infection with *R. parkeri* was recently published (17). It has also recently shown to be mildly pathogenic to guinea pigs (18). In our study, *R. parkeri* was the only detected SFG *Rickettsia* in *A. triste* ticks from Uruguay. Our data suggest that *A. triste* is a host of SFG *Rickettsia* in Uruguay, and *R. parkeri* could be the causative agent of human cases of rickettsioses in Uruguay.
